# Assessment attendance and treatment engagement with talking and internet-enabled therapies of people with and without a long-term physical health condition: analysis of Talking Therapies service data

**DOI:** 10.1192/bjo.2024.846

**Published:** 2025-02-19

**Authors:** Emma Jenkinson, Ruth A. Hackett, Rona Moss-Morris, Grace Wong, Jon Wheatley, Mirko Cirkovic, Joanna Hudson

**Affiliations:** Institute of Psychology Psychiatry and Neuroscience, King’s College London, UK; South London and Maudsley NHS Foundation Trust, London, UK; Homerton University Hospital NHS Foundation Trust, London, UK

**Keywords:** Adjustment disorders, depressive disorders, anxiety- or fear-related disorders, mental health services, primary care

## Abstract

**Background:**

Research indicates that treatment outcomes are poorer for people with long-term physical health conditions (LTCs) in Talking Therapies services (formerly known as Improving Access to Psychological Therapies). However, the impact of having an LTC on attendance at assessment and treatment appointments within Talking Therapies remains unclear. Internet-enabled therapies may be one way to overcome barriers to treatment engagement in Talking Therapies. However, their effect on engagement and the influence of LTC status on receipt of internet-enabled therapies is unknown.

**Aims:**

To explore the association between LTC status and assessment attendance, treatment engagement and internet-enabled therapy receipt within Talking Therapies services, and whether receipt of internet-enabled treatment bolsters engagement.

**Method:**

We used anonymous patient-level data from two inner London Talking Therapies services during January to December 2022 (*n* = 17 095 referrals). Binary logistic regression models were constructed to compare differences between LTC and non-LTC groups on (a) assessment attendance, (b) engagement and (c) internet-enabled therapy receipt. In our regression models, we controlled for key clinical and demographic covariates.

**Results:**

There were no differences between patients with or without an LTC in assessment attendance or treatment engagement, after controlling for covariates. Across the whole sample, receiving internet-enabled treatment increased engagement. People with an LTC were less likely to receive an internet-enabled treatment.

**Conclusions:**

Having an LTC does not negatively affect assessment attendance and engagement with talking therapies. However, receiving an internet-enabled treatment bolstered engagement in our regression models. People with an LTC were less likely to receive internet-enabled treatment.

The Improving Access to Psychological Therapies (IAPT) programme, now named the Talking Therapies programme, was introduced in England to provide equitable access to psychological therapy for people living with common mental health conditions such as depression and anxiety-related disorders (including, but not limited to, phobias, obsessive–compulsive disorder and post-traumatic stress disorder).^1,2^ These conditions affect approximately 16% of the population at any given time.^3^

National Health Service (NHS) Talking Therapies services are primary care services that provide psychological assessment and treatment to individuals in the general population who are experiencing common mental health conditions. In line with National Service Guidelines,^2^ eligible patients are expected to have clinically relevant depression (a score of ≥10 on the Patient Health Questionnaire-9 (PHQ-9)^4^) or anxiety (a score of ≥8 on the Generalised Anxiety Disorder-7 (GAD-7)^5^) for inclusion. However, this can vary based on individual service guidance. These services employ therapists typically trained in cognitive–behavioural therapy (CBT). Talking Therapies was founded based on the economic argument that increasing access to evidence-based psychological therapies would reduce cost burden to the NHS and welfare benefit system.^6^ In 2016, the Five Year Forward View policy document set out the need for the prioritisation of psychological support for people with long-term physical health conditions.^7^ Talking Therapy services use the abbreviation LTC to refer to long-term physical health conditions, which they define as conditions that currently cannot be cured and require ongoing management, such as cardiovascular disease, chronic obstructive pulmonary disease and diabetes.^8,9^ LTCs affect approximately 30% of the population, and people with LTC(s) are two to three times more likely to experience a mental health condition than those without an LTC.^10^ Co-occurring physical and mental health needs have been consistently associated with poorer clinical outcomes, reductions in quality of life and increased healthcare costs.^11–13^

In response to the Five Year Forward View,^7^ Talking Therapies services were commissioned to develop care pathways offering integrated mental and physical healthcare.^8^ However, treatment outcomes in the Talking Therapies programme are poorer for people with LTCs compared with their non-LTC counterparts.^14^ These findings remain despite the introduction of LTC care pathways within Talking Therapies services, although this evaluation was performed relatively soon after the LTC-specific guidance was introduced.^15^ Talking Therapies services are constantly refining the types of treatment interventions offered to people with LTCs to increase their efficacy. Indeed, two different Talking Therapies services have compared the efficacy of treatment interventions tailored to the needs of people with LTCs relative to a non-tailored standard treatment. Both services report larger treatment effects on depression and anxiety outcomes for people who accessed tailored LTC interventions.^16,17^ Pre–post analysis of a therapist-supported digital intervention developed specifically to treat distress in the context of LTCs implemented in routine Talking Therapies care reported large treatment effects on depression and anxiety outcomes for people with LTCs who were considered to have clinically significant baseline levels of depression and anxiety.^18^ Thus, despite poorer outcomes for people with LTCs in Talking Therapies services, treatments can be adapted for people with LTCs, which may enhance treatment effects.

## Usage outcomes in Talking Therapies

What remains unclear from the literature is whether LTC status affects a person’s ability to attend an assessment and remain continually engaged with treatment within Talking Therapies services. In addition, internet-enabled therapy is a treatment delivery method used by Talking Therapies. From April 2021 to March 2022, 648 617 sessions of internet-enabled therapy were reported, but whether this enhances or lessens engagement with treatment is unclear.^19^ Previous factors associated with attendance in Talking Therapies services indicate that being from a minority ethnic background,^20,21^ living in a more socially deprived area^20^ and coming to the services through a general practitioner (GP) referral rather than self-referral^20,22^ have been associated with reduced assessment and/or treatment attendance at Talking Therapies services. Additionally, higher depression scores at baseline and not meeting criteria for a common mental health disorder were risk factors for treatment non-engagement.^20^

We are aware of three studies that have examined the explanatory effect of LTC status on attendance at initial appointments within Talking Therapies services.^22–24^ One study showed that first appointment attendance was less likely to occur if a person had an LTC.^23^ The other studies showed no effect of LTC status on first appointment attendance.^22,24^ However, these studies did not consider the recorded purpose of the appointment in their analysis. Instead, one study^22^ looked at first appointment attendance only. The other study^24^ defined any attended appointment within the first contacts as an assessment. Two studies have examined the explanatory effect of LTC status on treatment attendance within Talking Therapies services.^24,25^ The first study^24^ showed that LTC status had no effect on attendance at treatment appointments. The authors defined treatment as any attended appointment after the first two appointments. In the second study,^25^ the authors did consider the appointment purpose. Here, treatment engagement was defined as attendance at two or more treatment sessions and a planned discharge status (e.g. ending treatment after agreement with a therapist). This study reported a statistically significant effect of LTC status on treatment engagement, but this only occurred when analyses were restricted to the cohort accessing treatment during the COVID-19 pandemic (2020). No statistically significant effect of LTC status was observed in this study when analyses were restricted to cohorts attending Talking Therapies before (2019) or after (2021) the pandemic.

These mixed findings are likely attributable to several factors, including the number of Talking Therapies sites included in the analyses, the way in which key variables such as attendance and engagement were defined, and the variables controlled for in statistical analyses. Additionally, differences in the time frames in which cohorts were studied may have also contributed (e.g. pre/post the implementation of the Talking Therapies LTC guidance, the COVID-19 pandemic). Thus, there remains the need to study the role of LTC status on assessment attendance and treatment engagement within Talking Therapies after the COVID-19 pandemic lockdown and the publication of the LTC guidelines. Further, considering the recorded purpose of the appointment may provide a more nuanced insight into the influence of LTC status on usage outcomes in Talking Therapies services.

## Internet-enabled therapies in Talking Therapies

In addition, the National Institute for Health and Care Excellence (NICE)^26^ recommend the delivery of therapy using remote delivery methods such as the telephone and internet. Indeed, research evidence suggests that remote therapies may bolster engagement with psychological therapies by overcoming barriers to attendance.^27,28^ Attendance may be a particular challenge for people with LTCs, because of multiple hospital appointments and mobility challenges associated with certain LTCs. However, a recent analysis of data from seven Talking Therapies services showed that people with an LTC were less likely to receive a telephone assessment compared with their non-LTC counterparts, despite a telephone delivered assessment being associated with higher attendance rates overall.^24^ LTC status had no effect on attendance at treatment appointments.^24^ However, irrespective of LTC status, people who were offered telephone-delivered treatment sessions were less likely to attend treatment.^24^ The potential of internet-enabled therapy treatment platforms, as one way to bolster treatment engagement and improve outcomes within the Talking Therapies programme, is recognised.^29^ However, the findings from the study by Saxon et al^24^ investigating telephone delivery raises questions as to whether the same patterns may be found for internet-enabled modalities, given the lack of face-to-face contact. To our knowledge, no studies have quantitatively explored the impact of internet-enabled therapy on engagement in Talking Therapies services and whether a person’s LTC status influences the mode of treatment received. Qualitative data from therapists working in Talking Therapies services^30^ suggests digital interventions may be perceived as a potential barrier to treatment engagement for people with LTCs, but this hypothesis is yet to be tested quantitatively.

## Aims

Therefore, the aims of this study were to use routinely collected data to investigate the following: (a) Does LTC status affect assessment attendance when controlling for potential demographic and clinical confounders? (b) Does LTC status and mode of treatment delivery affect attendance at two or more treatment sessions when controlling for potential demographic and clinical confounders? and (c) Does LTCs status affect a person’s likelihood of receiving internet-enabled therapy?

## Method

This paper is reported in accordance with the Strengthening the Reporting of Observational Studies in Epidemiology guidelines (STROBE).^31^ All procedures contributing to this work comply with the ethical standards of the relevant national and institutional committees on human experimentation and with the Helsinki Declaration of 1975, as revised in 2013. This research was part of an NHS Quality Improvement project, approval granted by London NHS Quality Improvement Board (signed off by the Director of Nursing at the relevant hospital Trusts on 7 January 2019 (service A) and 28 January 2022 (service B).

### Data

All data analysed in this study were collected as part of routine care for reporting to NHS Digital.^32^ NHS Talking Therapies services are required to collect data on all patients at the first attended contact and at each attended session thereafter, as part of the NHS Minimum Data Set. Outcome data includes scores on depression, anxiety and social functioning (see below for measures). Services also collect usage data such as the purpose, attendance and delivery modality of appointments, in line with the Talking Therapies Data Set (version 2.0).^32^ The current study analysed data from two inner London adult Talking Therapies providers between 1 January 2022 and 31 December 2022. Raw demographic and clinical data were collected from any individual referral to the two services during the stated time frame. It is possible that some individuals received more than one referral within the analysis time frame. All care episodes were included in the analysis, meaning that an individual could be included in the analysis more than once. The data used was anonymous and the research team had no contact with patients at this service.

### Participants

We included data from any individual aged 18 years or over with a referral to the included services over the 12-month period (*n* = 26 172). The sample was restricted to referrals with complete information on LTC status. Cases where LTC status was reported as ‘not stated’ were excluded from the sample. Patients who were still in active treatment were excluded for data completion reasons, and to avoid misclassifying a patient as not attending either an assessment or treatment when they have not had the opportunity to do so because they are still in active treatment. After missing and excluded cases were removed (*n* = 9077, 34.68%), a final sample of *n* = 17 095 remained (see Table 1). Verbal consent to data access was provided by participants and was recorded by healthcare professionals at the services; however, as the data was anonymous at the point of access, informed consent was not required. This is consistent with national guidelines for data reporting of Talking Therapies services to NHS Digital.^32^

**Table 1:** Demographic and clinical characteristics of final sample

Variable	Total sample, *n* (%)/mean (s.d.)	Non-LTC group, *n* (%)/mean (s.d.)	LTC group, *n* (%)/mean (s.d.)	Mean difference	95% CI	Statistical test	*P*-value
Baseline referrals	17 095	12 724 (74.43%)	4371 (25.57%)				
Age (years); *n* = 17 095	36.73 (12.74)	34.41 (11.01)	43.47 (14.85)	−9.06	−9.48 to −8.65	*t* = −42.6814	*P* < 0.001
Gender (% female); *n* = 16 911	11 735 (69.39%)	8661 (68.07%)	3074 (70.33%)			*χ* ^2^ = 7.5966	*P* = 0.006
Ethnicity; *n* = 16 176						*χ* ^2^ (4) = 113.7642	*P* < 0.001
Asian or Asian British	1227 (7.59%)	903 (7.10%)	324 (7.41%)				
Black or Black British	3483 (20.40%)	2341 (18.40%)	1142 (26.13%)				
Mixed	1330 (7.78%)	980 (7.70%)	350 (8.01%)				
Other	1161 (6.79%)	878 (6.90%)	283 (6.47%)				
White	8975 (52.50%)	6864 (53.95%)	2111 (48.30%)				
Minoritised ethnicity (% yes) *n* = 16 176	7201 (44.52%)	5102 (42.63%)	2099 (49.86%)			*χ* ^2^ (2) = 65.7280	*P* < 0.001
Deprivation decile; *n* = 16 991	3.39 (1.67)	3.46 (1.71)	3.19 (1.54)	0.27	0.21–0.33	*t* = 9.1835	*P* < 0.001
GP referral (% yes); *n* = 17 021	2040 (11.99%)	1317 (10.35%)	723 (16.54%)			*χ* ^2^ = 120.1492	*P* < 0.001
PHQ-9; *n* = 14 674	13.93 (6.19)	13.43 (6.14)	15.36 (6.13)	−1.93	−2.16 to −1.70	*t* = −16.6995	*P* ≤ 0.001
GAD-7; *n* = 14 669	12.52 (5.29)	12.26 (5.29)	13.24 (5.22)	−0.98	−1.18 to −0.79	*t* = −9.8788	*P* ≤ 0.001
WSAS; *n* = 13 459	18.14 (9.38)	17.55 (9.11)	19.92 (9.91)	−2.37	−2.74 to −2.01	*t* = −12.7970	*P* < 0.001
Service; % referred to service A	10 048 (58.78%)	7722 (60.69%)	2326 (53.21%)			*χ* ^2^ = 75.0079	*P* < 0.001
Attended assessment (% yes)	13 549 (79.26%)	10 003 (78.62%)	3546 (81.13%)				
Engaged (of those attended assessment, % yes)	4622 (34.11%)	3468 (34.67%)	1154 (32.54%)				
Internet-enabled therapy received (of those attended assessment, % yes)	861 (6.41%)	727 (7.27%)	141 (3.98%)				

LTC, long-term physical health condition; GP, general practitioner; PHQ-9, Patient Health Questionnaire-9; GAD-7, Generalised Anxiety Disorder-7; WSAS, Work and Social Adjustment Scale.

### Outcome variables

#### Objective 1: assessment attendance

Attendance at assessment was defined as cases with at least one contact with Talking Therapies where the purpose of the session was coded as ‘assessment’ or ‘assessment and treatment’, and it was attended (1 = attended assessment, 0 = did not attend assessment). The time frame for assessment attendance was restricted to appointments 1–4, based on service do not attend policies and the assumption that assessments will have been conducted within this time frame.

#### Objective 2: engagement with treatment

Engagement with treatment was defined as cases with two or more contacts with Talking Therapies where the purpose was coded as ‘treatment’ (1 = engaged, 0 = did not engage). It is assumed that cases where the purpose of the contact was ‘treatment’ have been deemed eligible for treatment after an assessment. The Talking Therapies manual defines people who have received two or more treatment sessions as having ‘completed treatment’.^2^ In this study, we have relabelled this as treatment engagement. The time frame for treatment engagement was pragmatically restricted to appointments 1–10, to manage the number of observations per case.

#### Objective 3: receipt of internet enabled therapy

We defined internet-enabled therapy as delivery of therapy through an internet-based programme, which is accessed by the person in their own time. This definition is consistent with the definition in the NHS data dictionary and Talking Therapies.^2,32^ Intervention modality is recorded by in-service clinicians during each contact meeting. It is possible for clinicians to select more than one intervention in one meeting. Because of this method of recording, we defined receipt of internet-enabled therapy as cases with at least one contact meeting where an internet-enabled intervention had been recorded by the clinician regardless of any other activity (1 = yes, 0 = no).

### Explanatory variables

#### LTC status

LTC status is a self-reported outcome collected at the point of (self)-referral or during the first contact appointment. In the current study, LTC status was defined as a binary variable (0 = LTC absent, 1 = LTC present).

#### Internet-enabled therapy receipt

Receipt of internet-enabled therapy was an explanatory variable only in the analysis where engagement was the outcome variable.

#### Covariates

Age, gender, ethnicity, social deprivation decile, Talking Therapies service, whether the participant had a GP referral and baseline clinical scores were included as covariates. Age (years), gender (0 = female, 1 = male) and ethnicity were self-reported and recorded at referral or assessment. Ethnicity categories were based on Office for National Statistics categories and were as follows: White; Black; Black British, Asian or Asian British; mixed ethnicity and other. These ethnicity categories were then collapsed into a binary variable for the main analyses (0 = White, 1 = minoritised ethnicity). We included data from two Talking Therapies services (0 = service A, 1 = service B). Referral source is routinely collected. For this study, GP referral source was derived as a binary variable (0 = not referred by GP, 1 = GP referral). Social deprivation decile was calculated based on postcode data. Participants’ postcodes were inputted into a publicly available government tool (English Indices of Deprivation 2019: Postcode Lookup (opendatacommunities.org)) that gives a deprivation rank based on the Lower Super Output Area (LSOA) that each postcode falls under.^33^ The Index of Multiple Deprivation decile is calculated by dividing the LSOAs into ten equal groups, with a lower decile indicating greater social deprivation. Baseline clinical scores are self-reported and collected routinely at the initial appointment, using three standardised questionnaires within the Talking Therapies programme: the PHQ-9,^4^ the GAD-7^5^ and the Work and Social Adjustment Scale (WSAS).^34^ The PHQ-9 is a nine-item depression questionnaire. Scores on the PHQ-9 range from 0 to 27; higher scores indicate greater depressive symptoms (a score of ≥10 indicates clinically relevant symptoms).^4^ The GAD-7 is a seven-item anxiety questionnaire. The GAD-7 has a scale range of 0–21; higher scores indicate greater anxiety symptoms (a score of ≥8 indicates clinically relevant symptoms).^5^ The WSAS measures functioning and is a five-item self-report questionnaire. The WSAS has a scale range of 0–40; higher scores indicate greater impairments in functioning.^34^

### Statistical analysis

Analyses were performed on Stata (version 17 for Windows). Descriptive and clinical characteristics of the sample were compared with *t*-tests for continuous variables and *χ* ^2^-tests for categorical variables by LTC status. Because of the consistent application of the data monitoring system within the Talking Therapies programme, cases of missing data were low. Therefore, any missing data were treated as blanks. To investigate the association between LTC status and (a) assessment attendance, (b) treatment engagement and (c) receipt of an internet-enabled therapy, binary logistic regression models were used to determine the relative contribution of LTC status to the three outcome variables. For the engagement logistic regression and internet-enabled therapy logistic regression, only participants who attended an assessment were included in the analyses. This was because patients cannot attend a treatment session or be offered an intervention before attending an assessment in Talking Therapies services. For each research objective, four regression models were tested. Analyses were run using complete cases. Model 1 was unadjusted. Model 2 was adjusted for age, gender, ethnicity and deprivation decile. Model 3 additionally included baseline PHQ-9 (depression), GAD-7 (anxiety) and WSAS (social functioning) scores, GP referral (no/yes) and Talking Therapies site (service A or B).

## Results

The demographic and clinical characteristics of the sample are shown in Table 1. Across the 17 095 participants the mean age was 36.73 years (s.d. = 12.74), 53.65% were female and over half (55.48%) were of White ethnicity. The average deprivation decile was 2.9, which suggests that the average participant lived in a postcode that was in the 10–20% most socially deprived areas in England. In our sample, 25.57% of participants identified as having an LTC. Participants with an LTC were significantly older on average, lived in more deprived areas, were more likely to be from a minoritised ethnic background and were more likely to be referred to Talking Therapies by their GP. They had significantly higher baseline depression (PHQ-9), anxiety (GAD-7) and social functioning (WSAS) scores compared with the non-LTC group (see Table 1 for details).

### Association of LTC status with outcomes

#### Attended assessment

The demographic and clinical characteristics of the sample who attended an assessment in Talking Therapies are shown in Supplementary Table 1. A total of 79.26% (*n* = 13 549) of participants attended a Talking Therapies assessment following a referral (*n* =17 095), and of these, 26.17% had an LTC and 73.82% did not. In models 1 and 2, people who reported having an LTC were significantly more likely to attend an assessment than those without (model 1: odds ratio 1.17, unadjusted; model 2: odds ratio 1.12, adjusted for sociodemographic characteristics) (see Table 2). When clinical characteristics and service site were entered into the model, no statistically significant effect of LTC status on assessment attendance was observed. Other statistically significant explanatory variables of assessment attendance indicated that individuals who were female (odds ratio 0.83; *P* = 0.011), from minoritised ethnic groups (odds ratio 0.82; *P* = 0.003) and those with better functioning (odds ratio 0.99; *P* = 0.040) were less likely to attend an assessment at Talking Therapies (see Supplementary Tables 3 and 4 for adjusted regression models). People who had higher baseline anxiety (odds ratio 1.02; *P* = 0.021) and were a patient within service A (odds ratio 4.29; *P* ≤ 0.001) were more likely to attend their assessment. Age, social deprivation, baseline depression scores or being referred by a GP did not significantly influence assessment attendance within our two services (see Supplementary Tables 3 and 4).

**Table 2:** Binary logistic regressions of long-term physical health condition status on assessment attendance, treatment engagement and receipt of internet-enabled therapies

Regression	Attended assessment			Engaged			Received internet-enabled therapy		
	Odds ratio	*P*-value	95% CI	Odds ratio	*P*-value	95% CI	Odds ratio	*P*-value	95% CI
Model 1^a^	1.17	<0.001	1.07–1.28	0.91	0.022	0.84–0.99	0.53	<0.001	0.44–0.64
Model 2^b^	1.12	0.018	1.02–1.24	0.94	0.141	0.86–1.02	0.67	<0.001	0.55–0.82
Model 3^c^	1.16	0.062	0.99–1.35	0.97	0.563	0.86–1.07	0.74	0.003	0.60–0.90

a. Model 1: unadjusted.

b. Model 2: adjusted for age, gender, ethnicity and deprivation decile.

c. Model 3: adjusted for age, gender, ethnicity, deprivation decile, baseline Patient Health Questionnaire-9 (depression), Generalised Anxiety Disorder-7 (anxiety) and Work and Social Adjustment Scale (social functioning) scores, general practitioner referral and service site.

#### Treatment engagement

Only 34.11% of those who attended an assessment in our sample (*n* = 13 549) went on to engage with treatment (*n* = 4622). Of these who engaged, 24.97% had an LTC and 75.03% did not. The demographic and clinical characteristics of the sample who engaged are presented in Supplementary Table 2. People with an LTC were statistically less likely to engage in treatment in the unadjusted model (odds ratio 0.91). However, when sociodemographic (model 2) and clinical characteristics and service site (model 3) were statistically accounted for, the effect of LTC status on treatment engagement was no longer statistically significant (see Table 2). Individuals from a minoritised ethnic background (odds ratio 0.81; *P* < 0.001), those with a higher baseline depression score (odds ratio 0.98; *P* < 0.001) and those referred by a GP (odds ratio 0.77; *P* < 0.001) were significantly less likely to engage with treatment. Individuals from a less socially deprived area (odds ratio 1.03; *P* = 0.010), those with a higher baseline anxiety score (odds ratio 1.03; *P* < 0.001) and those referred to service A (odds ratio 1.19; *P* < 0.001) were significantly more likely to engage in treatment. No other covariates were statistically significant explanatory variables of treatment engagement (see Supplementary Tables 3 and 4).

#### Internet-enabled therapy receipt

When investigating rates of internet-enabled therapy receipt in our sample who attended an assessment (*n* = 13 549), we found that 6.41% (*n* = 868) of patients received internet-enabled therapy at some point across their care pathway. Of those who received this, *n* = 141 (3.98%) had an LTC and *n* = 727 (7.27%) did not. See Supplementary Table 5 for the demographic and clinical characteristics of the sample who received internet-enabled treatment. Across all three models, LTC status was associated with a decreased likelihood of receiving internet-enabled therapy even after adjusting for demographic and clinical characteristics (odds ratio 0.74; *P* = 0.003) (see Table 2). Older patients (odds ratio 0.98; *P* < 0.001), those from a minoritised ethnic group (odds ratio 0.79; *P* = 0.003), those with poorer functioning (odds ratio 0.98, *P* = 0.002) and those referred to service B (odds ratio 0.16; *P* < 0.001) were significantly less likely to receive internet-enabled treatment (see Supplementary Tables 3 and 4).

### Effect of internet-enabled therapy receipt on treatment engagement

Across all three of our regression models, those who received internet-enabled therapy were significantly more likely to engage in treatment even after adjusting for demographic and clinical characteristics (see Table 3). Being older (odds ratio 1.00; *P* = 0.021), from a minoritised ethnic background (odds ratio 0.81; *P* < 0.001), being referred by a GP (odds ratio 0.76; *P* < 0.001) and having higher baseline depression scores (odds ratio 0.98; *P* < 0.001) and poorer social functioning scores (odds ratio 0.99; *P* = 0.005) predicted reduced likelihood of engagement irrespective of internet-enabled receipt. Having higher baseline anxiety scores (odds ratio 1.02; *P* < 0.001), lower deprivation scores (odds ratio 1.03; *P* < 0.001) and being referred to service A (odds ratio 1.30; *P* ≤ 0.001) were associated with increased likelihood of engagement irrespective of internet-enabled therapy receipt (See Supplementary Tables 6 and 7).

**Table 3:** Binary logistic regressions of receipt of internet-enabled therapies on treatment engagement

Regression	Engaged		
	Odds ratio	*P*-value	95% CI
Model 1^a^	2.95	<0.001	2.57–3.39
Model 2^b^	2.78	<0.001	2.40–3.21
Model 3^c^	2.68	<0.001	2.31–3.10

a. Model 1: unadjusted.

b. Model 2: adjusted for long-term physical health condition status, age, gender, ethnicity and deprivation decile.

c. Model 3: adjusted for long-term condition status age, gender, ethnicity, deprivation decile, baseline Patient Health Questionnaire-9 (depression), Generalised Anxiety Disorder-7 (anxiety) and Work and Social Adjustment Scale (social functioning) scores and general practitioner referral.

## Discussion

This study used patient data from two London adult NHS Talking Therapies services to explore potential variations in assessment attendance, treatment engagement and use of internet-enabled therapies among those with and without an LTC. Additionally, we explored whether use of internet-enabled therapies bolstered treatment engagement. Our findings indicate that individuals with an LTC were no more likely to attend an assessment appointment than those without an LTC when we controlled for demographic factors, clinical factors and site. Likewise, LTC status did not influence engagement after controlling for covariates. Individuals offered an internet-enabled therapy were more likely to engage in treatment, but people with LTCs were less likely to receive this treatment method compared with those without an LTC.

We found that LTC status did not affect rates of assessment appointment attendance. This is consistent with previous quantitative evidence^22,24^ showing that LTC status does not influence initial appointment attendance. This lack of association is promising for Talking Therapies services as it suggests that once referred, these services are successfully supporting LTC clients to attend their assessment. A hypothesised reason for this lack of association is that services are likely responding well to LTC guidelines,^8^ such as uptake of LTC-specific training. This may support clinicians to better engage people with LTCs. Additionally, remote delivery (e.g. telephone or videoconferencing) forms a key part of Talking Therapies services^2^ and can reduce potential physical barriers to attendance. This may decrease the practical barriers faced by people with LTCs and generate more equitable access. Hence, consistent with other studies,^25^ this may be another reason for the lack of association with LTC status. Individuals who were referred to service A were significantly more likely to attend their assessment, with large effect. This is likely because of the variability between services based on their location, culture and commissioning. We can only speculate reasons for this effect; however, one reason may be that Talking Therapies services have different links with local organisations for signposting patients to access support within the community, thus influencing assessment non-attendance in Talking Therapies services. Providing information to patients and referrers on local organisations that could offer support outside of Talking Therapies services may help people to contact services that are appropriate for their needs.

Having an LTC was not significantly associated with treatment engagement in our study when service, demographic and clinical factors were controlled for. This suggests that engagement with treatment sessions did not vary by LTC status. This may suggest that the association between LTC status and increased engagement seen during the COVID-19 pandemic^25^ was related to factors other than LTC status. We hypothesise that one reason for this contrasting finding may have been the heightened levels of mental health difficulties, specifically for people with LTCs.^35^ Additionally, as hypothesised for assessment attendance, a transition to remote appointments (i.e. face-to-face therapy delivered via videoconferencing) may have removed additional practical barriers to engagement for people with an LTC. Our findings are encouraging as they suggest that efforts by Talking Therapies services to engage patients, including those with LTCs, have been successful at reducing potential inequalities.

Our study demonstrated that people with an LTC had a significantly reduced likelihood of receiving an internet-enabled therapy compared with people without an LTC, yet receipt of internet-enabled therapies improved engagement. Internet-enabled therapies are effective for people with LTCs.^18,36^ However, qualitative evidence suggests that patient^18,37^ and therapist views^30^ on the value of digital treatment are mixed. It has been indicated that Talking Therapies clinicians feel that internet-enabled treatment is acceptable for people with LTCs and may be advantageous for engagement.^30^ However, referral patterns to internet-enabled therapy across the two sites in our study did not mirror this pattern, and suggest that potential biases may be in place that are preventing referrals to internet-enabled treatment for LTC clients. The ‘digital divide’ is well established and postulates that some demographic groups, including older adults and those from minority ethnic groups, may be disproportionally excluded from using digital treatment because of reduced confidence, skill and/or access to digital products.^38^ Our findings correspond with this as people who were older and those from a minority ethnic group were significantly less likely to receive internet-enabled treatment (regardless of LTC status). However, our findings remained after controlling for these demographic factors, suggesting that other LTC-specific barriers may influence this relationship over and above these factors.

From the data available to us, it is unclear if differences in internet-enabled intervention receipt are a result of people with an LTC declining to uptake internet-enabled treatment or that this treatment option was not considered by clinicians. However, other work has indicated that clinicians feel less confident explaining the role of psychological therapy within LTCs,^30^ which may explain the negative association between LTC status and internet-enabled intervention receipt. This may be because of perceived complexities such as increased physical health demands. Hence, assessing clinicians may default to other options such as LTC-specific groups, rather than digital treatment. Therefore, more work may be needed to educate clinicians on the value of internet-enabled treatments as a suitable and effective option for people living with an LTC. It has been suggested that speciality LTC roles, such as ‘LTC Champions’, increase patient engagement within Talking Therapies services and provide support for non-LTC clinicians;^39^ therefore, Talking Therapies commissioners should aim to develop and promote these roles.

We found that across all patients, those who received an internet-enabled intervention at some point across their care pathway were more likely to engage with treatment with moderate effect. This was irrespective of LTC status. To our knowledge, this is the first study to quantitatively investigate if internet-enabled therapies bolster engagement within Talking Therapies settings. Qualitative studies have shown that patients identify benefits in internet-enabled therapies, including flexibility, convenience and accessibility,^18^ potentially accounting for our findings in part. Additionally, internet-enabled therapies have been shown to be effective for treatment outcomes in within Talking Therapies services for patients with^18^ and without LTCs.^40^ This suggests the benefits of internet-enabled treatment to both patients and services could be two-fold. However, because of issues with current reporting, we were unable to quantify the dose of internet-enabled therapy receipt within our study, and therefore more work is needed to explore this.

It is well established that internet-enabled treatment is one way in which Talking Therapies services can scale up delivery and provide accessible, evidence-based and potentially cost-effective^40^ psychological therapies. Current evidence indicates that outcomes are poorer for people with an LTC than those without an LTC within Talking Therapies services.^15^ Therefore, internet-enabled treatments may be a solution to improve this and support engagement for LTC clients. Consistent work shows that LTC-specific CBT protocols with greater therapeutic relevance produce better treatment outcomes^16,18,41^ and increase engagement.^39^ Hence, Talking Therapies guidance recommend that treatment protocols are adapted for people with an LTC.^8^ Despite this guidance, qualitative work suggests that this still remains a challenge for therapists within Talking Therapies, because of service- and system-level constraints.^30,42^ Digital treatments may be one way in which services can provide more tailored support to patients in a cost-effective manner. Additionally, through using an internet-enabled modality such as a guided support platform, clinician burden may be reduced. A real-world implementation study has indicated that digital therapist-guided interventions, tailored to the needs of those with LTCs, may be effective and acceptable to patients when delivered in Talking Therapies care.^18^

It is important to note that overall, under a quarter of patients went on to engage with treatment, irrespective of LTC status and intervention modality in our study. This has clinical implications for Talking Therapies services that future research should aim to address.

### Strengths and limitations

To our knowledge, this is the first study to explore the influence of having an LTC on usage outcomes in Talking Therapies services in a post-COVID-19 lockdown cohort. The use of NHS real-world Talking Therapies data in this study provides insight into these outcomes in current clinical practice. Additionally, through considering the recorded purpose of appointments, we were able to distinguish between attendance at assessments and treatment sessions. This allows for a more nuanced understanding of usage outcomes across the treatment pathway. Further, we were able to include people that may usually be underrepresented in traditional research, such as those from diverse ethnic and social backgrounds. We explored the representativeness of our sample, compared with those of the local population, using the latest UK Census data.^43^ This suggested that our sample is largely representative of the local population served by the NHS Talking Therapies services in our study, based on ethnicity and age. Specifically, just over 40% of our sample were from minority ethnic groups, and this largely reflects the ethnic breakdown of the population served: 47% and 48%, respectively, for the Talking Therapies services in our sample. Further, the mean age of our sample (36 years) was slightly lower than that of the weighted mean age of the populations they served (41 years), but this younger referral age aligns with that seen in previous published work.^44^ However, the use of data from inner London services makes it difficult to generalise to a broader UK context. In this study, we relied on self-reported LTC diagnoses and treated it as a binary variable, which has been done in other studies that used Talking Therapies data.^10^ However, the use of self-reported LTC status may present inaccuracies in our analysis. Additionally, missing data on LTC status and the exclusion of patients without data means that we were unable to capture all referrals. Further we selected cut-offs for our outcome variables. We restricted assessment attendance to the first four appointments and treatment engagement to the first ten Talking Therapies appointments. These restrictions were pragmatically and clinically selected, considering service do not attend policies and data management challenges. However, these restrictions may present a level of bias in our analyses. Additionally, the missing data present for baseline clinical outcomes may also present biases in our analyses. We were unable to report reasons for non-assessment attendance, non-engagement and intervention receipt in our study, and therefore more work should be done to explore these reasons. Indeed, the reporting of some of the variables within the current data-set was reliant on practitioner and administrator accuracy, thus some uncertainties surrounding the reliability of the reporting remain.

In conclusion, there were no differences between people with and without an LTC in assessment attendance or treatment engagement once we controlled for key confounders. Across the whole sample, receiving internet-enabled treatment bolstered engagement. However, people with an LTC were less likely to receive internet-enabled treatment.

## Supporting information

Jenkinson et al. supplementary material Jenkinson et al. supplementary material

## Data Availability

The data used in the study are available on request from the corresponding author, J.H. The data are not publicly available as they could compromise the privacy of the participants.

## Supplementary material

The supplementary material is available online at https://doi.org/10.1192/bjo.2024.846.
