# Supplementary material for: Assessment attendance and treatment engagement with talking and internet-enabled therapies of people with and without a long-term physical health condition: analysis of Talking Therapies service data
Source: BJPsych Open. 2025 Feb 19;11(2):e72. doi: 10.1192/bjo.2024.846 (PMC12052583; doi:10.1192/bjo.2024.846)
Supplement: Jenkinson et al. supplementary material [file S2056472424008469sup001.docx]

Supplementary Table 1: The demographic and clinical characteristics of those who attended an assessment.

|  | n(%)/mean (sd) | Non-LTC group | LTC group | Mean diff | 95% CI | Statistical test | *p*-value |
| --- | --- | --- | --- | --- | --- | --- | --- |
| Total attended assessment: | 13,549 | 10,003 (73.82%) | 3546 (26.17%) |  |  | *X*^2=^12.47 | *p*<.001 |
| ***Demographic variables*** |  |  |  |  |  |  |  |
| Age (years): | 36.74 (12.61) | 34.33 (10.70) | 43.53 (14.89) | -9.20 | -9.66 to -8.74 | t=-39.443 | *p*<.001 |
| Gender (% Female) | 9,288 (69.30%) | 6,802(68.74%) | 2,486 (70.87%) |  |  | *X*^2=^0.5.49 | *p*=0.019 |
| Ethnicity:  Asian or Asian British  Black or black British  Mixed  Other  White | 978 (7.54%)  2666 (20.56 %)  1,018 (7.85%)  931 (7.18%)  7,377 (56.88%) | 716 (7.16%)  1,769 (17.68%)  738 (7.38%)  704 (7.04%)  5,610 (56.10%) | 262 (7.39%)  897 (25.30%)  280 (7.90%)  227 (6.40%)  1,767 (49.83%) |  |  | *X*^2^=97.25 | *p*<.001 |
| Ethnic minority (% Yes)  Missing: n= 600 | 5593 (43.19%) | 3927 (39.26%) | 1666 (46.98%) |  |  | *X*^2^=57.73 | *p*<.001 |
| Deprivation decile: | 3.40 (1.68) | 3.47 (1.73) | 3.17 (1.53) | 0.30 | 0.24 to 0.36 | t= 9.13 | *p*<.001 |
| GP referral (% Yes) | 1,486 (11.00%) | 913 (9.13%) | 573 (16.16%) |  |  | *X*^2^=134.13 | *p*<.001 |
| Talking Therapies service, A | 7,665 (56.57%) | 5,825 (58.23%) | 1,840 (51.89%) |  |  | *x*^2^ = 42.87 | *p*<0.001 |
| ***Clinical variables*** |  |  |  |  |  |  |  |
| PHQ-9 | 13.88 (6.17) | 13.36 (6.11) | 15.33 (6.20) | -1.97 | -2.20 to -1.73 | *t*= -16.35 | *p*<.001 |
| GAD-7 | 12.51 (5.27) | 12.25 (5.27) | 13.2 (5.19) | -0.98 | -1.18 to -0.78 | *t* =-9.48 | *p*<.001 |
| WSAS | 18.05 (9.32) | 17.44 (9.06) | 19.85 (9.85) | -2.41 | -2.79 to -2.03 | *t*= -12.50 | *p*<.001 |

CI= Confidence Interval; LTC= Long-term Condition.

Supplementary Table 2: The demographic and clinical characteristics of those who engaged with treatment.

|  | n(%)/mean (sd) | Non-LTC group | LTC group | Mean diff | 95% CI | Statistical test | *p*-value |
| --- | --- | --- | --- | --- | --- | --- | --- |
| Total Engaged: | 4622 | 3,468 (34.67%) | 1,154 (32.54%) |  |  | *X*^2=^1.204 | *p=0.022* |
| ***Demographic variables*** |  |  |  |  |  |  |  |
| Age (years): | 36.63 (12.04) | 34.49 (10.14) | 43.06 (14.71) | -8.57 | -9.33 to -7.81 | t= -22.02 | *p*<.001 |
| Gender (% Female) | 3222 (70.27%) | 2410 (70.10%) | 812 (70.79%) |  |  | *X*^2=^1.99 | *p*=0.656 |
| Ethnicity:  Asian or Asian British  Black or black British  Mixed  Other  White | 291 (6.30%)  850 (18.40%)  337 (7.29%)  267 (5.78%)  2,758 (59.67%) | 209 (6.03%)  550 (15.86%)  245 (7.06%)  202 (5.82%)  2,171 (62.60%) | 82 (7.12%)  300 (26.00%)  92 (7.97%)  65 (5.63%)  587 (50.87%) |  |  | *X*^2^(4) =70.56 | *p*<.001 |
| Ethnic minority (% Yes): | 1,745 (37.75%) | 1,206 (34.77%) | 539 (46.70%) |  |  | *X*^2^=52.58 | *p*< .001 |
| Deprivation decile: | 3.49 (1.75) | 3.58 (1.81) | 3.21 (1.55) | 0.37 | 0.26 to 0.49 | t=6.27 | *p*<.001 |
| GP referral: (% Yes): | 401 (8.68%) | 242 (6.98%) | 159 (13.77%) |  |  | *X*^2^=50.59 | *p*<.001 |
| Talking Therapies service, A: (% Yes): | 2,682 (58.03%) | 2,068 (59.63%) | 614 (53.21%) |  |  | *x*^2^ =14.67 | *p*<0.001 |
| ***Clinical variables*** |  |  |  |  |  |  |  |
| PHQ-9 | 13.62 (5.7) | 13.14 (5.6) | 15.07(5.75) | -1.93 | -2.31 to -1.55 | *t*= -10.0 | *p*<.001 |
| GAD-7 | 12.67 (4.88) | 12.48 (4.85) | 13.23 (4.94) | -0.76 | -1.08 to -0.43 | *t* =-4.56 | *p*<.001 |
| WSAS | 17.84 (8.65) | 17.27 (8.32) | 19.56 (9.40) | -2.29 | -2.88 to -1.71 | *t*= -7.75 | *p*<.001 |

CI= Confidence Interval; LTC= Long-term Condition

Supplementary table 3: Binary logistic regressions of long-term condition status on assessment attendance, treatment engagement, and receipt of internet-enabled therapies (Model 2)

| Model 2 | Attended assessment | | | Engagement | | | Internet-enabled therapy | | |
| --- | --- | --- | --- | --- | --- | --- | --- | --- | --- |
|  | Odds ratio | P-value | 95% CI | Odds ratio | P-value | 95% CI | Odds ratio | P-value | 95% CI |
| LTC status | 1.12 | 0.018 | 1.02 -1.24 | 0.94 | 0.141 | 0.86 -1.02 | 0.67 | <0.001 | 0.56 - 0.82 |
| Age | 1.00 | 0.456 | 1.00- 1.00 | 1.00 | 0.548 | 1.00 -1.00 | 0.98 | <0.001 | 0.97-0.99 |
| Gender | 0.97 | 0.458 | 0.89-1.05 | 1.06 | 0.176 | 0.97-1.14 | 0.90 | 0.199 | 0.68 – 0.92 |
| Ethnicity | 0.76 | <0.001 | 0.71-0.83 | 0.77 | <0.001 | 0.71-0.83 | 0.79 | 0.002 | 0.59-0.86 |
| Deprivation decile | 0.99 | 0.960 | 0.98-1.03 | 1.04 | <0.001 | 1.02-1.07 | 1.13 | <0.001 | 1.09-1.17 |

CI= confidence interval; LTC= long-term condition. Variables included in Model 2: LTC status, age, gender, ethnicity, and deprivation decile.

Supplementary table 4: Fully adjusted binary logistic regressions of long-term condition status on assessment attendance, treatment engagement, and receipt of internet-enabled therapies (Model 3)

| Model 3 | Attended assessment | | | Engagement | | | Internet-enabled therapy | | |
| --- | --- | --- | --- | --- | --- | --- | --- | --- | --- |
|  | Odds ratio | P-value | 95% CI | Odds ratio | P-value | 95% CI | Odds ratio | P-value | 95% CI |
| LTC status | 1.16 | 0.062 | 0.99 -1.35 | 0.97 | 0.563 | 0.89- 1.07 | 0.74 | 0.003 | 0.60 - 0.90 |
| Age | 1.00 | 0.441 | 1.00- 1.01 | 1.00 | 0.135 | 1.00 -1.01 | 0.98 | <0.001 | 0.97 - 0.98 |
| Gender | 0.83 | 0.011 | 0.72-0.96 | 1.02 | 0.602 | 0.94-1.11 | 0.92 | 0.337 | 0.79 – 1.09 |
| Ethnicity | 0.82 | 0.003 | 0.70-0.94 | 0.81 | <0.001 | 0.75-0.87 | 0.79 | 0.003 | 0.68 – 0.92 |
| Deprivation decile | 1.02 | 0.202 | 0.99-1.06 | 1.03 | 0.010 | 1.01-1.05 | 1.02 | 0.457 | 0.98-1.06 |
| PHQ-9 | 0.99 | 0.091 | 0.97-1.00 | 0.98 | <0.001 | 0.97-0.99 | 0.98 | 0.035 | 0.97-1.00 |
| GAD-7 | 1.02 | 0.021 | 1.00-1.04 | 1.03 | <0.001 | 1.02-1.04 | 1.02 | 0.096 | 1.00-1.04 |
| WSAS | 0.99 | 0.040 | 0.98-1.00 | 1.00 | 0.467 | 0.99-1.00 | 0.98 | 0.002 | 0.97-0.99 |
| GP referral | 0.99 | 0.891 | 0.81-1.21 | 0.77 | <0.001 | 0.67-0.88 | 0.99 | 0.918 | 0.76-1.27 |
| Service | 4.29 | <0.001 | 3.59-5.14 | 1.19 | <0.001 | 1.10-1.29 | 0.16 | <0.001 | 0.13-0.20 |

CI= confidence interval; LTC= long-term condition. Variables included in model 3: LTC status, age, gender, ethnicity, deprivation decile, baseline PHQ-9 (depression), GAD-7 (anxiety) and WSAS (social functioning) scores, GP referral source and service.

|  | n(%)/mean (sd) | Non-LTC group | LTC group | Mean diff | 95% CI | Statistical test | *p*-value |
| --- | --- | --- | --- | --- | --- | --- | --- |
| Total Attended assessment: | 13,549 | 10,003 | 3,546 |  |  |  |  |
| Total received internet-enabled therapy (% yes of those attended assessment): | 868 (6.41%) | 727 (7.27%) | 141 (3.98%) |  |  | *X*^2^ =47.30 | *p*<.001 |
| ***Demographic variables*** |  |  |  |  |  |  |  |
| Age (years): | 33.35 (9.80%) | 32.82 (9.31) | 36.16 (11.42) | -3.34 | -5.12 to -1.56 | t= -3.69 | *p*<.001 |
| Gender (% Female) | 620 (71.84%) | 520 (71.82%) | 100 (71.94%) |  |  | *X*^2=^0.0008 | *p*=0.977 |
| Ethnicity:  Asian or Asian British  Black or black British  Mixed  Other  White | 47 (5.41%)  143 (16.47%)  49 (5.65%)  63 (7.26%)  519 (59.79%) | 35 (4.81%)  108 (14.86%)  38 (5.26%)  57 (7.84%)  449 (61.76%) | 12 (8.51%)  35 (24.82%)  11 (7.80%)  6 (4.48%)  70 (4.26%) |  |  | *X*^2^(4) = 16.41 | *P=* *0.003* |
| Ethnic minority (% Yes): | 310 (35.71%) | 244 (33.56%) | 66 (46.81%) |  |  | *X*^2^= 8.29 | *p*=0.004 |
| Deprivation decile: | 3.80 (1.88) | 3.83(1.89) | 3.60 (1.80) | 0.22 | -0.12 0.56 | t= 1.29 | *p=* 0.198 |
| GP referral: (% Yes): | 84 (9.68%) | 65 (8.94%) | 19 (13.48%) |  |  | *X*^2^= 2.76 | *P=* 0.097 |
| Talking Therapies service A: (% Yes): | 775 (89.29%) | 662 (91.06%) | 113 (80.14%) |  |  | *x*^2^ =14.71 | *p*<0.001 |
| ***Clinical variables*** |  |  |  |  |  |  |  |
| PHQ-9 | 12.92 (5.79) | 12.81 (5.80) | 13.51 (5.90) | -0.71 | -1.77 to 0.35 | *t*= - 1.31 | *p=*0.095 |
| GAD-7 | 12.25 (5.13) | 12.15 (5.14) | 12.77 (5.08) | -0.62 | - 1.56 to 0.32 | *t* =-1.29 | *p=* 0.098 |
| WSAS | 17.84 (8.65) | 16.57 (8.05) | 18.43 (8.94) | -1.90 | -3.40 to -0.36 | *t*= -7.75 | *p=*0.008 |

Supplementary Table 5: The demographic and clinical characteristics of those who received internet-enabled therapy.

Supplementary table 6: Binary logistic regressions of binary logistic regressions of receipt internet-enabled therapies on treatment engagement (Model 2)

| Model 2 | Engagement | | |
| --- | --- | --- | --- |
|  | Odds ratio | P-value | 95% CI |
| Internet enabled therapy | 2.78 | <0.001 | 2.40- 3.21 |
| LTC status | 0.95 | 0.310 | 0.87 -1.04 |
| Age | 1.00 | 0.789 | 0.99- 1.00 |
| Gender | 1.05 | 0.231 | 0.97-1.14 |
| Ethnicity | 0.78 | <0.001 | 0.72-0.84 |
| Deprivation decile | 0.92 | <0.001 | 0.90-1.15 |

CI= confidence interval; LTC= long-term condition. Variables included in Model 2: LTC status, age, gender, ethnicity, and deprivation decile.

Supplementary table 7: Fully adjusted binary logistic regressions of receipt internet-enabled therapies on treatment engagement (Model 3)

| Model 3 | Engagement | | |
| --- | --- | --- | --- |
|  | Odds ratio | P-value | 95% CI |
| Internet-enabled therapy | 2.68 | <0.001 | 2.30 – 3.10 |
| LTC status | 0.99 | 0.807 | 0.90 -1.09 |
| Age | 1.00 | 0.021 | 1.00- 1.01 |
| Gender | 1.02 | 0.681 | 0.94-1.11 |
| Ethnicity | 0.81 | <0.001 | 0.75-0.88 |
| Deprivation decile | 1.03 | 0.013 | 1.01 -0.05 |
| PHQ-9 | 0.98 | <0.001 | 0.97-0.99 |
| GAD-7 | 1.02 | <0.001 | 1.02-1.05 |
| WSAS | 0.99 | 0.737 | 0.99-1.00 |
| GP referral | 0.76 | <0.001 | 0.66-0.88 |
| Service | 1.30 | <0.001 | 1.20-1.41 |

CI= confidence interval; LTC= long-term condition. Variables included in model 3: LTC status, age, gender, ethnicity, deprivation decile, baseline PHQ-9 (depression), GAD-7 (anxiety) and WSAS (social functioning) scores, GP referral source and IAPT service.
